# Effect of Liraglutide on Vascular Inflammation Evaluated by [^64^Cu]DOTATATE

**DOI:** 10.3390/diagnostics11081431

**Published:** 2021-08-08

**Authors:** Emilie H. Zobel, Rasmus S. Ripa, Bernt J. von Scholten, Viktor Rotbain Curovic, Lars Jorge Diaz, Tine W. Hansen, Peter Rossing, Andreas Kjaer

**Affiliations:** 1 Steno Diabetes Center Copenhagen, Niels Steensens Vej 2, 2820 Gentofte, Denmark; bjos@novonordisk.com (B.J.v.S.); viktor.rotbain.curovic@regionh.dk (V.R.C.); lars.jorge.diaz@regionh.dk (L.J.D.); tine.willum.hansen@regionh.dk (T.W.H.); peter.rossing@regionh.dk (P.R.); 2 Department of Clinical Physiology, Nuclear Medicine & PET and Cluster for Molecular Imaging, Rigshospitalet and University of Copenhagen, 1165 Copenhagen, Denmark; rasmus.ripa@regionh.dk (R.S.R.); akjaer@sund.ku.dk (A.K.); 3 Novo Nordisk A/S, 2860 Søborg, Denmark; 4 Department of Clinical Medicine, University of Copenhagen, 1165 Copenhagen, Denmark

**Keywords:** GLP-1 RA, vascular inflammation, PET, type 2 diabetes

## Abstract

Quantification of vascular inflammation before and after treatment with glucagon-like peptide-1 receptor agonists (GLP-1 RAs) may help reveal mechanistic pathways underlying the cardiovascular benefits of these drugs. We assessed change in vascular inflammation in the carotid arteries over 26 weeks by copper-64-labeled [1,4,7,10-tetraazacyclododecane-N,N′,N″,N‴-tetraacetic acid]-D-Phe1, Tyr3-octreotate ([^64^Cu]DOTATATE) PET in 30 participants included in a substudy of a double-blind trial where persons with type 2 diabetes (T2D) were randomized to liraglutide (*n* = 15) or placebo (*n* = 15) for 26 weeks. Mean age (SD) was 66.4 (7.2) years, HbA_1c_ 56.4 (9.2) mmol/mol and BMI 28.9 (4.6) kg/m^2^. Weight and HbA_1c_ were significantly reduced by liraglutide vs. placebo (*p* ≤ 0.01). The [^64^Cu]DOTATATE uptake (mean standardized uptake values) was significantly reduced in the liraglutide-treated group (−0.11 [95% confidence interval −0.19 to −0.03], *p* = 0.01) and not changed significantly in the placebo group (−0.07 [−0.14 to 0.01], *p* = 0.08). The mean difference between groups did not reach significance (−0.04 [−0.15 to 0.07], *p* = 0.44). In conclusion, [^64^Cu]DOTATATE uptake was reduced in persons with T2D treated with liraglutide. However, the reduction compared to placebo did not reach statistical significance, perhaps due to limited power. A reduction in vascular inflammation with liraglutide could help explain the cardiovascular protection observed with GLP-1 RAs in outcome studies but warrants further and larger studies.

## 1. Introduction

Inflammation is a key process in progressive atherosclerosis and quantification of vascular inflammation in atherosclerosis may reveal mechanistic pathways for drugs with the ability to reduce the risk of cardiovascular disease. The majority of clinical research aimed at in vivo evaluation of vascular inflammation has used fluorine-18-labeled fluorodeoxyglucose positron emission tomography ([^18^F]FDG PET). A large body of evidence links the [^18^F]FDG uptake to the abundance of macrophages in atherosclerotic plaques, including studies of excised human plaques [1]. Carotid [^18^F]FDG uptake has also been demonstrated to identify a reduction in vascular inflammation in response to statin treatment [2]. Lack of cell specificity is one limitation of [^18^F]FDG imaging, which has fueled interest in more cell-specific probes, including copper-64-labeled [1,4,7,10-tetraazacyclododecane-N,N′,N″,N‴-tetraacetic acid]-D-Phe1, Tyr3-octreotate ([^64^Cu]DOTATATE). [^64^Cu]DOTATATE targets the G-protein-coupled receptor somatostatin receptor subtype-2 (SST2) that is selectively expressed on the surface of activated macrophages. Preclinical [3] and retrospective studies [4,5] suggest that DOTATATE binding is a specific marker of macrophage activity, and a study in 10 persons undergoing endarterectomy demonstrated that [^64^Cu]DOTATATE accumulates in atherosclerotic plaques of the carotid artery [6]. No clinical trials have yet evaluated change in vascular inflammation using [^64^Cu]DOTATATE PET [6] and head-to-head comparisons of the FDG and DOTATATE PET tracer are limited to a single study [7]. 

Several large cardiovascular outcome trials have demonstrated that glucagon-like peptide-1 receptor agonists (GLP-1 RAs) reduce the risk of cardiovascular disease in type 2 diabetes [8,9,10,11]. Animal studies have demonstrated that GLP-1 RAs reduce atherosclerotic plaque formation [12,13] and affect key steps in the development of atherosclerotic plaques, including macrophage-derived foam cell formation [14] and the adhesion of mononuclear cells to the endothelium [15]. We therefore conducted a randomized placebo-controlled clinical trial to evaluate the GLP-1 RA liraglutide’s effect on vascular inflammation. 

We found no effect of liraglutide on the primary endpoint, which was change in vascular inflammation assessed using [^18^F]FDG PET in 102 participants, however, an exploratory analysis indicated a possible effect of liraglutide on [^18^F]FDG uptake in the subgroup of participants with a history of cardiovascular disease [16].

Here, we present a substudy of this trial aimed to investigate liraglutide’s effect on vascular inflammation, evaluated as [^64^Cu]DOTATATE uptake in the carotid arteries, in a subgroup of 30 participants.

## 2. Materials and Methods

### 2.1. Study Design and Participants

The main trial included 102 participants with type 2 diabetes in a double-blind, randomized controlled clinical setting, as previously described in detail [16]. In short, participants were randomized in a 1:1 ratio to treatment with liraglutide or placebo for 26 weeks. Starting dose was 0.6 mg once daily for 1 week, followed by 1.2 mg once daily for 1 week, followed by 1.8 mg once daily for the remainder of the trial. The dose escalation was flexible, and participants were kept on the highest tolerated dose. 

Major inclusion criteria were age > 50 years; HbA_1c_ ≥ 48 mmol/mol (6.5%); and eGFR ≥ 30 mL/min/1.73 m^2^ (estimated by the CKD-EPI formula). Glucose- and cholesterol-lowering treatment had to be stable for min. 4 weeks prior to enrollment in the study. Major exclusion criteria were type 1 diabetes; other treatment (90 days prior to enrollment in the study) or disorders (except for conditions associated with type 2 diabetes history), which could interfere with the results of the trial. The full list of inclusion and exclusion criteria has previously been published [16].

Here, we report results from a prespecified substudy in 30 participants where vascular inflammation was evaluated by both [^18^F]FDG uptake and [^64^Cu]DOTATATE uptake. The number of included participants in this substudy was limited by [^64^Cu]DOTATATE tracer availability, and inclusion was random. [^18^F]FDG PET/CT imaging was performed at baseline and after 26 weeks of liraglutide/placebo treatment. [^64^Cu]DOTATATE PET/CT imaging was performed on a separate day, preferably within the same week as [^18^F]FDG PET/CT imaging, but timing was pragmatic due to limited [^64^Cu]DOTATATE tracer availability. The median [IQR] interval between the 2 imaging studies was 8 [6,7,8,9,10,11,12,13,14] days for the baseline examination and 6 [2,3,4,5,6,7,8,9,10,11,12,13,14,15] days for the follow-up examination

The study was approved by the local ethics committee (H-16044546) and the Danish Medicines Agency (2016110109) and was in compliance with the principles of the Declaration of Helsinki. All participants provided informed consent.

### 2.2. Blood and Urine Analysis

HbA_1c_ was measured using high-performance liquid chromatography. Plasma creatinine was measured by an enzymatic method (Hitachi 912, Roche Diagnostics, Mannheim, Germany) and to calculate eGFR we used the CKD-EPI equation [17]. Urinary albumin creatinine rate (UACR) was measured by an enzyme immunoassay in two consecutive morning urine samples. High-sensitivity C-reactive protein was measured in lithium–heparin–plasma using Cobas 8000 e801 (Roche Diagnostics, Rotkreuz, Switzerland) assays. 

### 2.3. [^64^Cu]DOTATATE PET/CT

We used a combined PET/CT scanner (Siemens Biograph mCT64, Siemens, Berlin, Germany). Patients were scanned one hour (±10 min) after [^64^Cu]DOTATATE injection. The PET was acquired in three-dimensional list mode for 10 min centered at the carotid bifurcation. A low-dose CT scan (120 keV, mAs 50) was applied for attenuation correction and anatomical location of the carotid arteries. The PET images were reconstructed using CT-based attenuation correction, with both resolution–recovery (point spread function, TrueX) and time-of-flight (2 iterations, 21 subsets, zoom 1.0) giving 400 × 400 image slices (voxel size 2.00 × 2.04 × 2.04). A 2 mm full-width-at-half-maximum Gaussian filter was then applied. 

The PET quantification was carried out using OsiriX MD 11.0 (Pixmeo, Bernex, Switzerland). The baseline and follow-up examinations were analyzed in parallel to ensure correct alignment between timepoints, however, the reader was blinded to the order of the examinations. The carotid arteries were identified and traced with freehand or ellipse regions of interest (ROIs) on the axial CT slices without use of the PET images. Afterwards, the ROIs were copied onto the spatially aligned PET examination. The carotids were traced from 2 cm proximal to the bifurcation and as distal as the internal carotid artery was identifiable on the non-contrast enhanced CT. 

For each ROI, we quantified the DOTATATE uptake as the standardized uptake value (SUV) by measuring a maximum pixel activity value (SUVmax) and mean pixel activity (SUVmean). For sensitivity, we also calculated target-to-background ratio (TBR) as a ratio of SUVmax and the average blood SUV estimated from venous blood in the superior cava vein or the jugular vein.

### 2.4. [^18^F]FDG PET/CT

Details of the procedure are described in [16]. In brief, [^18^F]FDG-PET/CT imaging of the carotid arteries was undertaken two hours (±15 min) after injection of 4 MBq/kg 18F-FDG. Prior to injection of [^18^F]FDG, patients fasted for six hours. Insulin-treated patients could eat a small meal up to two hours before the examination, however, fast-acting insulin was not to be taken in the two hours before the examination. Before injection of the [^18^F]FDG, blood glucose was measured. Patients were instructed to rest lying in a bed between injection and scanning. There was no limit on exercise prior to the visit. Mean standardized uptake value (SUV) of [^18^F]FDG was measured in the carotids using an approach similar to the [^64^Cu]DOTATATE PET/CT. PET/CT images were analyzed by a masked, experienced reader.

### 2.5. Statistical Analysis

No formal power calculation was carried out for this substudy. The sample size for the entire study was based on the FDG PET/CT imaging. We aimed at including as many participants from the main study as possible and the number of participants in the substudy was determined by tracer availability at the time of inclusion in the main study. By chance, we ended up including 15 participants from each treatment group.

We present normal distributed data as the mean (standard deviation (SD)), continuous-scale non-normal distributed data (diabetes duration, urinary albumin creatinine ratio, high-sensitivity C-reactive protein (hsCRP)) as the median (interquartile range (IQR)) and categorical variables as numbers and percentages. An unpaired *t*-test and χ^2^ test or Fisher’s exact test, as appropriate, were used to find differences in clinical characteristics between treatment groups at baseline. The continuous-scale non-normal distributed variables were log2 transformed in all analyses. A paired *t*-test was used to compare baseline and end-of-treatment values within groups and an unpaired *t*-test to compare the change from baseline to end-of-treatment between treatment groups. We used linear regression to analyze the correlation between [^64^Cu]DOTATATE and [^18^F]FDG PET uptake and provide R^2^ to present the correlation between the two, and the F-test was applied to determine whether this relationship was statistically significant.

*p* < 0.05 was considered statistically significant. All statistical analyses were performed using SAS software (version 9.4; SAS Institute, Cary, NC, USA).

## 3. Results

### 3.1. Patients

We included 30 participants in this substudy, 15 treated with liraglutide and 15 with placebo. Baseline characteristics (Table 1) were as follows: mean age (SD) 66.4 (7.2) years; median [IQR] diabetes duration 12.3 [5.7–19.8] years; mean HbA_1c_ 56.4 (9.2) mmol/mol; mean eGFR 85.8 (13.3) mL/min/1.73m^2^; and 8 (26.7%) had a history of cardiovascular disease. Baseline characteristics were well balanced between the liraglutide and the placebo treated group, except for triglycerides, which were higher in the liraglutide group (*p* = 0.01). The baseline characteristics for the participants included in the substudy were comparable to the 72 participants only included in the main study (Table 2).

Mean change in HbA_1c_ was −6.1 mmol/mol (95% confidence interval (CI) −10.1 to −2.1) in the liraglutide-treated group compared to −0.1 mmol/mol (−2.6 to 2.4) in the placebo group, resulting in a mean difference of −6.1 mmol/mol (−10.6 to −1.5) between groups (*p* = 0.01). Mean change in body weight was −3.0 kg (−4.7 to −1.2) in the liraglutide-treated group compared to −0.2 kg (−1.0 to 0.65) in the placebo group, resulting in a mean difference of −2.8 kg (−4.7 to −0.90) between groups (*p* = 0.006). Mean change in LDL cholesterol was −0.26 mmol/L (−0.48 to −0.05) in the liraglutide-treated group compared to −0.29 mmol/L (−0.50 to −0.07) in the placebo-treated group, with a mean difference of 0.02 mmol/L (−0.27 to 0.31) between groups (*p* = 0.88). Mean change in systolic blood pressure was 6.7 mmHg (1.8 to 11.6) in the liraglutide-treated group compared to 3.1 mmHg (−4.2 to 10.4) in the placebo group, with a mean difference of 3.5 mmHg (−4.9 to 12.0) between groups (*p* = 0.40). The level of hsCRP was unchanged in both groups, with no difference between groups (*p* = 0.51).

### 3.2. [^64^Cu]DOTATATE Uptake in Carotid Arteries

**Figure 1:**
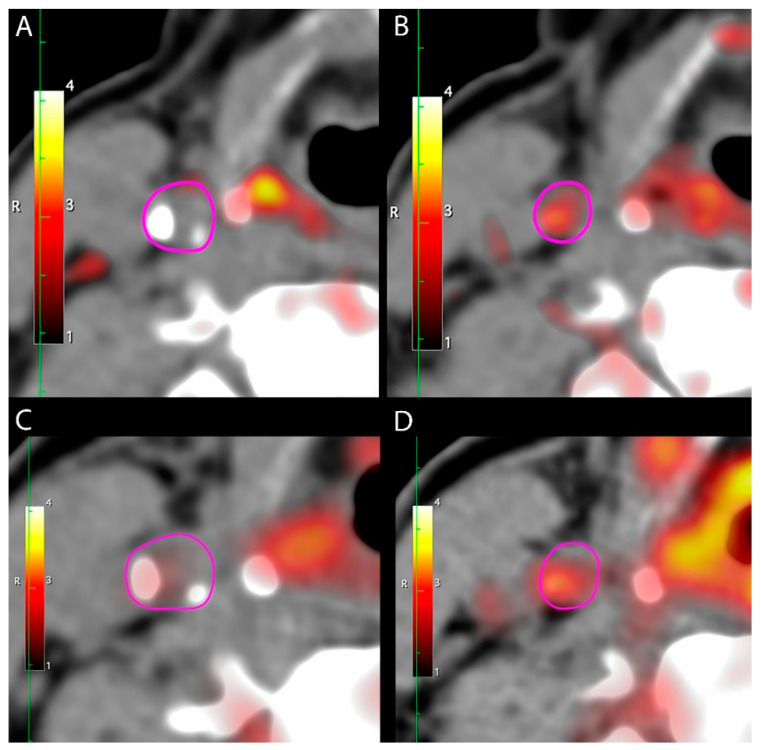
PET/CT images at baseline with high and low uptake of [^64^Cu]DOTATATE. Representative baseline axial images showing the carotid artery outlined in pink. (**A**). An axial slice with calcifications and low uptake of [^64^Cu]DOTATATE (SUV_mean_ = 0.9). (**B**) An axial slice with minimal calcifications and high uptake of [^64^Cu]DOTATATE (SUV_mean_ = 1.4). (**C**,**D**) [^18^F]FDG uptake in the same locations for comparison.

**Figure 2:**
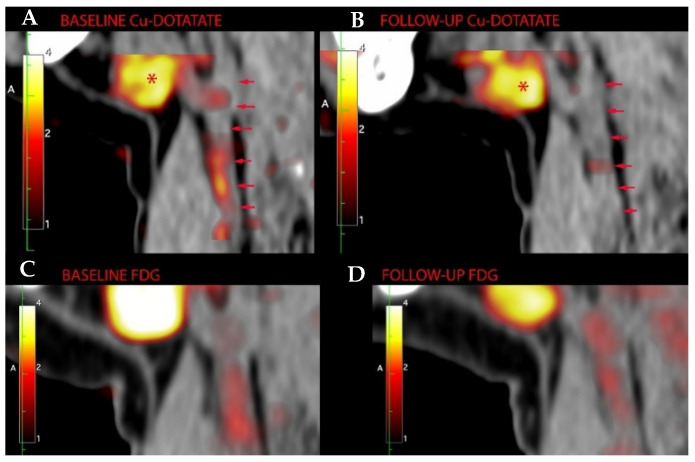
Sagittal [^64^Cu]DOTATATE-PET/CT images from a participant treated with liraglutide. The left carotid artery is outlined at (**A**) baseline (SUV_mean_ = 1.2) and (**B**) follow-up examination (SUV_mean_ = 0.92). * Physiological uptake in submandibular gland. The lower panels show [^18^F]FDG uptake from the same participant for comparison at (**C**) baseline and (**D**) follow-up.

**Figure 3:**
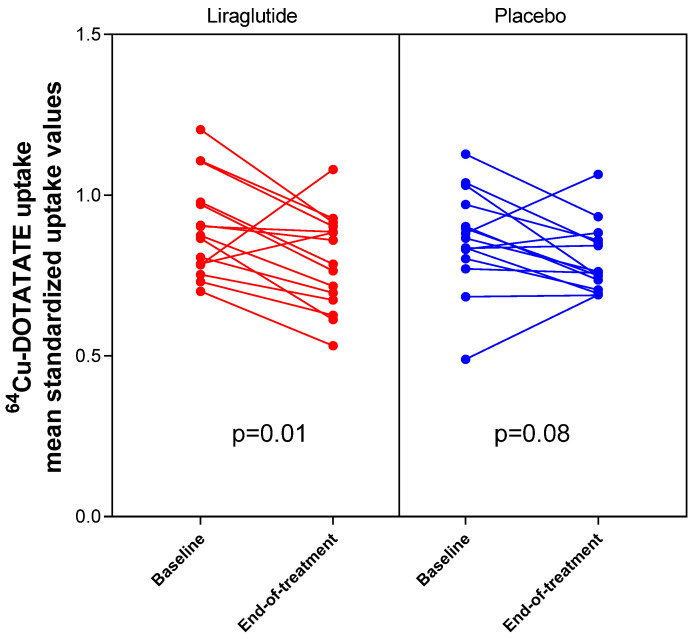
Effect of liraglutide and placebo on vascular inflammation evaluated with [^64^Cu]DOTATATE PET in the carotid arteries. [^64^Cu]DOTATATE uptake evaluated as mean standardized uptake values at baseline and end-of-treatment for the individual participants. Paired *t*-test was used to compare baseline and end-of-treatment values within groups.

Vascular inflammation in the carotid arteries was evaluated as change in [^64^Cu]DOTATATE uptake assessed as change in SUV_mean_ from baseline to end-of-treatment (Figure 1, Figure 2 and Figure 3, Table 3). We observed a significant decrease in SUV_mean_ in the liraglutide-treated group (−0.11 [95% CI −0.19 to −0.03] *p* = 0.01) and no significant change in SUV_mean_ in the placebo group (−0.07 [−0.14 to 0.01], *p* = 0.08). The mean difference between change in SUV_mean_ in the two groups did not reach statistical significance (−0.04 [−0.15 to 0.07], *p* = 0.44). We confirmed these findings when we assessed [^64^Cu]DOTATATE uptake as change in SUV_max_ (liraglutide-treated group: mean change in SUV_max_ −0.15 [−0.27 to −0.03] *p* = 0.02; placebo-treated group: mean change in SUV_max_ −0.09 [−0.20 to 0.02], *p* = 0.11; mean difference between groups: −0.06 [−0.22 to 0.10], *p* = 0.45). There was no difference between groups when assessing [^64^Cu]DOTATATE uptake as change in TBR (liraglutide-treated group: mean change 0.20 [−0.05 to 0.46] *p* = 0.11; placebo-treated group: mean change 0.14 [−0.15 to 0.43], *p* = 0.32; mean difference between groups: 0.06 [−0.31 to 0.43], *p* = 0.73).

### 3.3. [^64^Cu]DOTATATE Uptake vs. [^18^F]FDG Uptake in Carotid Arteries 

The vascular inflammation in the carotid arteries evaluated as change in [^18^F]FDG uptake from baseline to end-of-treatment was unchanged (SUV_mean_ in the liraglutide-treated group: −0.04 [95% CI −0.12 to 0.04], *p* = 0.28 and in the placebo group: −0.06 [−0.16 to 0.04], *p* = 0.25; without difference between groups 0.01 [−0.11 to 0.14], *p* = 0.83). 

**Figure 4:**
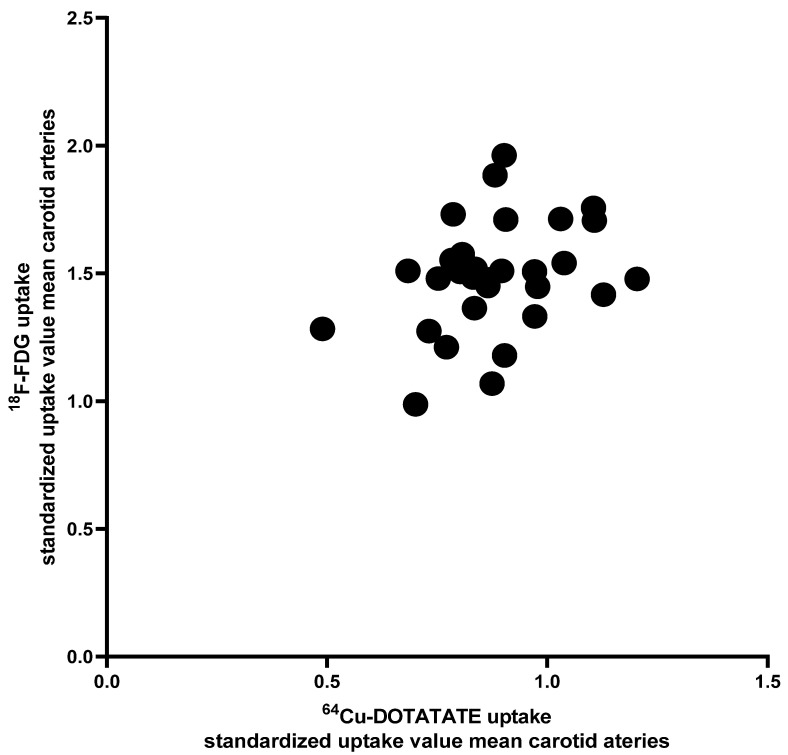
Correlation between [^64^Cu]DOTATATE and [^18^F]FDG PET uptake evaluated as mean standardized uptake value in the carotid arteries. Linear regression analysis revealed R^2^ = 0.11 and no correlation between the two measures (*p* = 0.07, evaluated by F-test).

At baseline, the correlation between [^64^Cu]DOTATATE and the [^18^F]FDG uptake in the carotid arteries evaluated as SUV_mean_ was small and without statistical significance (R^2^ = 0.11, *p* = 0.07, Figure 4). The change in uptake from baseline to end-of-treatment assessed with these two tracers was also not correlated (R^2^ = 0.06, *p* = 0.20). 

## 4. Discussion

In a substudy of a randomized placebo-controlled clinical trial, we demonstrated that vascular inflammation assessed using [^64^Cu]DOTATATE was reduced over 26 weeks in the 15 participants treated with liraglutide. In the 15 participants receiving placebo, the [^64^Cu]DOTATATE uptake was unchanged. However, the difference in vascular inflammation between the two treatment groups did not reach statistical significance, possibly due to lack of power. To the best of our knowledge, this is the first study to assess vascular inflammation using [^64^Cu]DOTATATE in an intervention trial. Our findings hint at a mechanistic pathway, which could contribute to the cardiovascular benefit observed with GLP-1 RAs in outcome studies. 

The interest in the effect of GLP-1 RAs on vascular inflammation is fueled by an increasing amount of preclinical and, to a lesser extent, clinical data that point toward an anti-inflammatory effect of these agents [18]. Preclinical data, primarily in mice, have demonstrated that GLP-1 RAs inhibit the formation of atherosclerotic plaques [12,19], suppress macrophage-derived foam cell formation in the plaques [14] and decrease the adhesion of mononuclear cells to the vessel wall [15]. This effect on atherosclerotic plaque formation requires the endothelial GLP-1 receptor [20] and seems partly independent of a reduction in weight and cholesterol [13]. Clinical data have demonstrated that liraglutide treatment reduces the levels of the inflammatory marker sCD163 and inflammatory macrophages [21] and one study demonstrated reduced inflammation in carotid plaques in patients treated with GLP-1-based therapy who had undergone endarterectomy [22].

As recently published, we observed no effect of liraglutide on arterial [^18^F]FDG uptake in the main LiraFlame study including 102 participants [16]. [^18^F]FDG PET has been successfully used to assess the impact of pharmaceutical interventions on arterial inflammation in a number of clinical trials [2,23,24], and several studies over the last decade have shown that [^18^F]FDG PET can be used to quantify arterial inflammation reliably and noninvasively [25,26,27]. Sadeghi et al. has reviewed a number of small animal and clinical studies that have investigated the biological link between arterial inflammation and the [^18^F]FDG signal, and several of these studies have demonstrated a correlation between [^18^F]FDG uptake and in vivo macrophage markers, but without indications that inflammation is the main determinant of arterial [^18^F]FDG uptake [28]. This is supported by a recent study in minipigs demonstrating that [^18^F]FDG uptake in medial smooth muscle cells was a significant contributor to the arterial [^18^F]FDG signal [29]. Overall, the participants in the LiraFlame study had a low level of vascular inflammation at baseline, and therefore the specificity of the tracer could be detrimental in order to detect subtle changes in vascular inflammation induced by liraglutide. 

The [^64^Cu]DOTATATE tracer is a novel marker of vascular inflammation, and binds specifically to somatostatin receptor-2 expressed by activated inflammatory macrophages. This specificity potentially provides significant advantage for detecting subtle changes in vascular biology and therefore represents an interesting supplement to the [^18^F]FDG tracer when evaluating effects on vascular inflammation [7]. Only a few studies have used somatostatin receptor imaging in atherosclerosis, and most have used the [^68^Ga]DOTATATE tracer. The difference between labeling with ^68^Ga and ^64^Cu mainly relates to substantially better spatial resolution using ^64^Cu due to the physical properties of the isotopes. When studying inflammation in small structures, such as the carotid artery wall, this difference in spatial resolution may be of utmost importance.

The two studies that have used the [^64^Cu]DOTATATE tracer have demonstrated an association between [^64^Cu]DOTATATE uptake and the Framingham risk score [5] and [^64^Cu]DOTATATE uptake in carotid atherosclerotic plaques in patients undergoing endarterectomy and markers of macrophages measured ex vivo [6].

We observed that liraglutide reduced vascular inflammation assessed as [^64^Cu] DOTATATE uptake, both when evaluated as SUVmean and SUVmax, but not as TBR. Even though the difference between the liraglutide- and placebo-treated group did not reach statistical significance, we consider this finding to be relevant in terms of explaining the cardiovascular protection observed with GLP-1 RAs. This is because (1) we revealed a signal in only 15 participants and (2) this signal was comparable to the signal observed in the main LiraFlame study, where the [^18^F]FDG uptake was reduced in participants with a history of cardiovascular disease (*n* = 23). This subgroup also had a higher level of vascular inflammation at baseline, providing the possibility to detect a change in [^18^F]FDG PET, whereas [^64^Cu]DOTATATE PET with higher specificity could also sense a signal in persons with lower levels of vascular inflammation. 

Given that the existing evaluation of [^64^Cu]DOTATATE in human vascular inflammation is sparse, we also evaluated the correlation between the [^64^Cu]DOTATATE and the [^18^F]FDG uptake in the carotid arteries and observed only a small correlation without statistical significance, underscoring that uptake of these two tracers probably reflects different components of vascular inflammation. 

The main strengths of this study are the randomized placebo-controlled design and the use of a novel high-resolution PET tracer to specifically quantify macrophages in vascular inflammation and their response to treatment. The primary limitation is the small number of included patients in the substudy. Another limitation is the patient population. Liraglutide has been shown to reduce cardiovascular events in patients with established cardiovascular disease, but in this study, we included an unselected type 2 diabetes population and only eight of the 30 participants had established cardiovascular disease.

## 5. Conclusions

In conclusion, our study demonstrated a significant decrease in [^64^Cu]DOTATATE uptake in the carotid arteries in persons with type 2 diabetes treated with liraglutide. However, the difference between the liraglutide- and placebo-treated group did not reach statistical significance. A potential reduction in vascular inflammation with liraglutide could help explain the cardiovascular protection observed with GLP-1 RAs in outcome studies but warrants further and larger studies.

## Figures and Tables

**Table 1:** Characteristics of the participants at baseline.

	Total (*n* = 30)	Liraglutide (*n* = 15)	Placebo (*n* = 15)	*p* Value
Sex (Women)	5 (16.7%)	2 (13.3%)	3 (20.0%)	1.0
Age (years)	66.4 (7.2)	65.9 (8.3)	66.9 (6.3)	0.69
Body mass index (kg/m^2^)	28.9 (4.3)	29.5 (4.0)	28.2 (4.7)	0.41
Type 2 diabetes				
Known duration of DM (years)	12.3 [5.7–19.8]	13.9 [5.9–20.9]	8.8 [5.5–17.2]	0.43
HbA_1c_ (mmol/mol)	56.4 (9.2)	59.1 (10.4)	53.7 (7.0)	0.11
Kidney function				
Estimated glomerular filtration rate (mL/min/1.73 m^2^)	85.8 (13.3)	86.2 (14.4)	85.4 (12.6)	0.88
Urinary albumin creatinine ratio (mg/g)	5.5 [4.5–12.5]	5.5 [2.5–12.5]	5.5 [5.0–14.5]	0.74
Cardiovascular risk factors				
Systolic blood pressure (mmHg)	134 (19)	133 (14)	136 (24)	0.68
LDL cholesterol (mmol/L)	2.2 (0.51)	2.2 (0.54)	2.1 (0.49)	0.70
Triglycerides (mmol/L)	1.7 (0.88)	2.1 (0.97)	1.3 (0.55)	0.01
Current smoker	1 (3.3%)	1 (6.7%)	0 (0%)	1.0
Hypertension	22 (73.3%)	12 (80.0%)	10 (66.7%)	0.68
High-sensitivity C-reactive protein (mg/L)	1.4 [0.9–3.1]	1.4 [1.0–3.3]	2.0 [0.8–2.8]	0.88
History of cardiovascular disease *	8 (26.7%)	6 (40.0%)	2 (13.3%)	0.21
Glucose-lowering medication				
Insulin use	12 (40.0%)	8 (53.3%)	4 (26.7%)	0.14
SGLT2 inhibitors	6 (20.0%)	3 (20.0%)	3 (20.0%)	1.0
Cardiovascular medication				
Aspirin treatment	10 (33.3%)	4 (26.7%)	6 (40.0%)	0.44
Lipid-lowering treatment	27 (90.0%)	14 (93.3%)	13 (86.7%)	1.0

Data are *n* (%), mean (SD) or median [IQR]. Hypertension was defined as treatment with anti-hypertensive medication. * A history of cardiovascular atherosclerotic disease was defined as a history of acute myocardial infarction, percutaneous coronary intervention, coronary artery bypass graft, stroke, peripheral arterial thrombosis, claudication and/or nitroglycerin-requiring angina pectoris. DM: diabetes mellitus, SGLT2: sodium glucose transporter 2.

**Table 2:** Participants in the substudy compared to participants only included in the main study.

	Included in Substudy (*n* = 30)	Not Included in Substudy (*n* = 72)	*p*-Values
Sex (Women)	5 (16.7%)	11 (15.3%)	1.0
Age (years)	66.4 (7.2)	66.4 (8.6)	1.0
Body mass index (kg/m^2^)	28.9 (4.3)	30.3 (4.7)	0.15
Type 2 diabetes			
Known duration of DM (years)	12.3 [5.7–19.8]	10.6 [5.7–18.2]	0.30
HbA1c (mmol/mol)	56.4 (9.2)	57.0 [52.0-64.0]	0.20
Kidney function			
Estimated glomerular filtration rate (mL/min/1.73 m^2^)	85.8 (13.3)	82.1 (17.3)	0.30
Urinary albumin creatinine ratio (mg/g)	5.5 [4.5–12.5]	6.3 [3.5–16.0]	0.30
Cardiovascular risk factors			
Systolic blood pressure (mmHg)	134 (19)	136 (17)	0.61
LDL cholesterol (mmol/L)	2.2 (0.51)	2.1 (0.73)	0.48
Triglycerides (mmol/L)	1.7 (0.88)	1.9 (1.1)	0.33
Current smoker	1 (3.3%)	13 (18.1%)	0.06
Hypertension	22 (73.3%)	57 (79.2%)	0.52
HsCRP (mg/L)	1.4 [0.9–3.1]	1.6 [0.86–4.2]	0.10
History of cardiovascular disease **	8 (26.7%)	15 (20.8%)	0.52
Glucose-lowering medication			
Insulin use	12 (40.0%)	27 (37.5%)	0.81
SGLT2 inhibitors	6 (20.0%)	14 (19.4%)	0.95
Cardiovascular medication			
Aspirin treatment	10 (33.3%)	27 (37.5%)	0.69
Lipid-lowering treatment ***	27 (90.0%)	61 (84.7%)	0.75

Data are n (%), mean (SD) or median [IQR]. Hypertension was defined as treatment with anti-hypertensive medication. ** A history of cardiovascular atherosclerotic disease was defined as a history of acute myocardial infarction, percutaneous coronary intervention, coronary artery bypass graft, stroke, peripheral arterial thrombosis, claudication and/or nitroglycerin-requiring angina pectoris. *** Lipid-lowering treatment defined as statins or ezetimibe. DM: diabetes mellitus, SGLT2: sodium glucose transporter 2.

**Table 3:** Carotid inflammation evaluated with PET in 30 persons with type 2 diabetes.

	Baseline Mean (SD)	End-of-Treatment Mean (SD)	*p* Value	∆ (95% CI)	*p* Value
[^64^Cu]DOTATATE uptake					
Carotid SUV_mean_					
Liraglutide, *n* = 15	0.90 (0.15)	0.79 (0.15)	0.01	−0.11 (−0.19; −0.03)	0.44
Placebo, *n* = 15	0.86 (0.15)	0.80 (0.11)	0.08	−0.07 (−0.14; 0.01)	
[^18^F]FDG uptake					
Carotid SUV_mean_					
Liraglutide, *n* = 15	1.4 (0.24)	1.4 (0.23)	0.28	−0.04 (−0.12; 0.04)	0.83
Placebo, *n* = 15	1.5 (0.20)	1.5 (0.21)	0.25	−0.06 (−0.16; 0.04)	

Vascular inflammation evaluated as mean standardized uptake values (SUV_mean_). Data are mean (SD) or change (95% CI). Paired *t*-test for comparisons between baseline and end-of-treatment within groups and unpaired *t*-test for comparison of the change from baseline to end-of-treatment between the two groups.

## Data Availability

The data presented in this study are available on reasonable request from the corresponding author. The data are not publicly available due to risk of re-identification.
